# Supplementary material for: METHimpute: imputation-guided construction of complete methylomes from WGBS data
Source: BMC Genomics. 2018 Jun 7;19:444. doi: 10.1186/s12864-018-4641-x (PMC5992726; doi:10.1186/s12864-018-4641-x)
Supplement: Supplementary file 1 — Figures S1-S12. (PDF 1832 kb) [file 12864_2018_4641_MOESM1_ESM.pdf]

Supplementary Figures

METHimpute: Imputation-guided construction of complete methylomes from WGBS data

Aaron Taudt, David Roquis, Amaryllis Vidalis, René Wardenaar, Frank Johannes and Maria Colomé-Tatché

Supplementary Figure 1

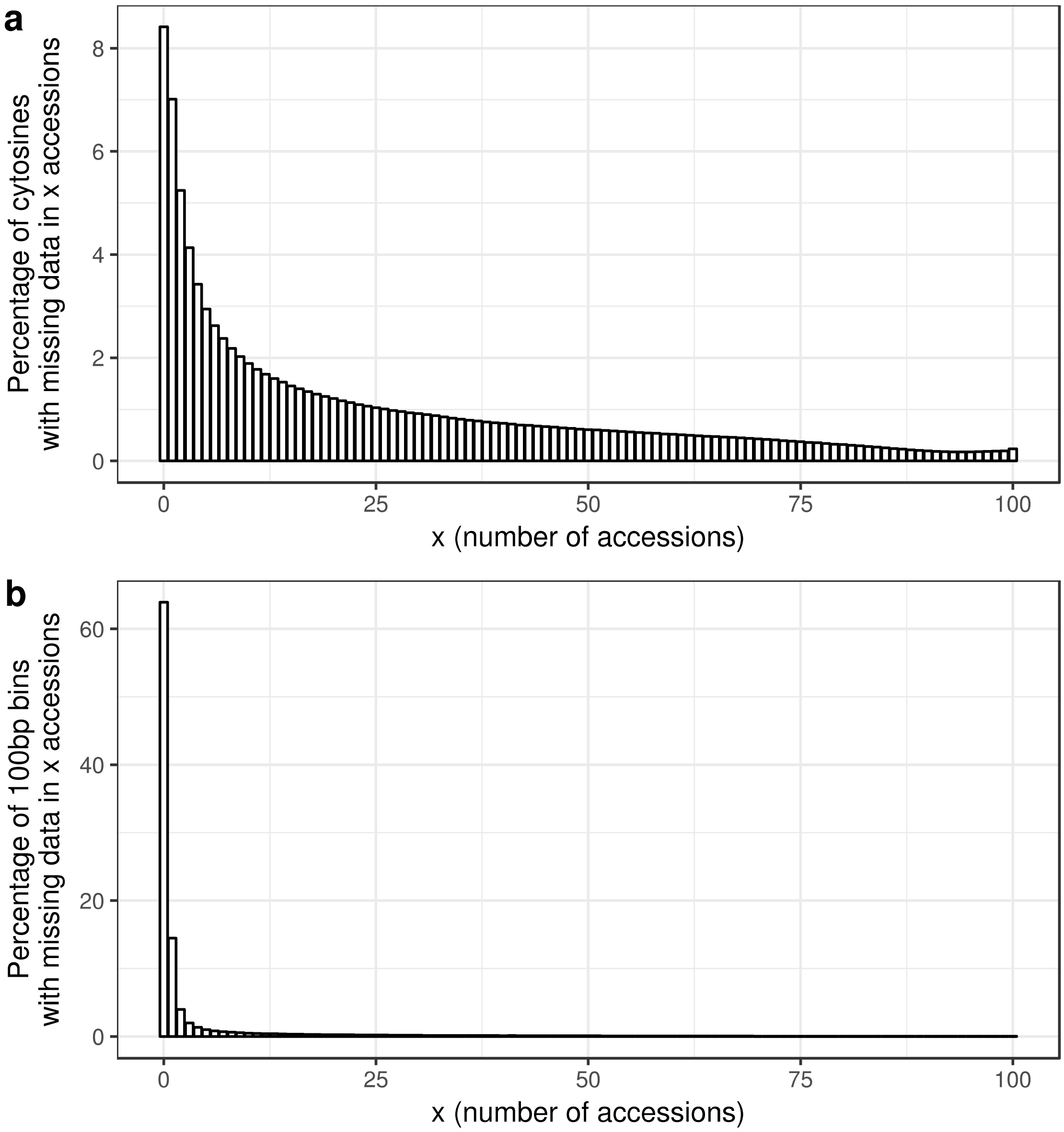

Figure SI-1: Missing cytosines in population epigenetic studies. For certain applications in population epigenetic studies (*e.g.* meQTL, mSFS, epimutation rates), only positions that are covered in all samples can be used. This leads to substantial dropout of usable positions if the number of samples is high. The y-axis shows the percentage of all **(a)** cytosines and **(b)** 100bp bins that are not covered (zero reads) in x samples. For example, in (a) ~ 8% of cytosines have missing data in 0 samples, meaning that only 8% of cytosines are covered in all samples, while 92% are missing in at least one sample. The data for this graph is from the 1001 methylomes project [1]. Mean coverage of this study was 5X (per strand and cytosine).

Supplementary Figure 2

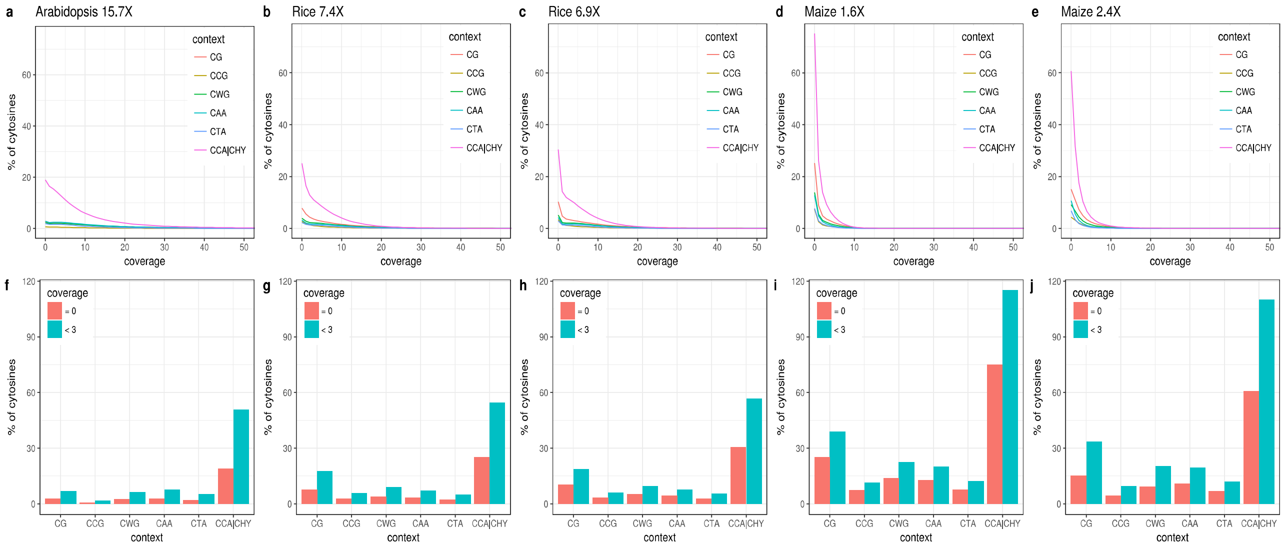

Figure SI-2: Coverage distributions for replicates. (a-e) Percentage of cytosines with X coverage (strand-specific). **(f-j)** Percentage of cytosines with missing data (red) and "uninformative" coverage (green), defined as less than three reads.

Supplementary Figure 3

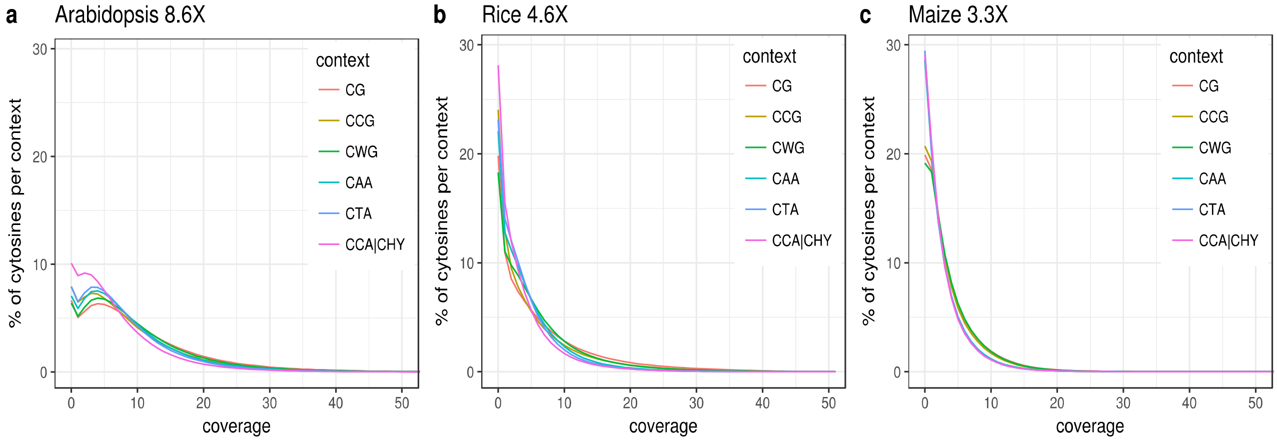

Figure SI-3: Context-specific coverage distributions. Percentage of cytosines with X coverage (strand-specific), normalized by context. The CHH context has a higher percentage of missing and uninformative cytosines.

Supplementary Figure 4

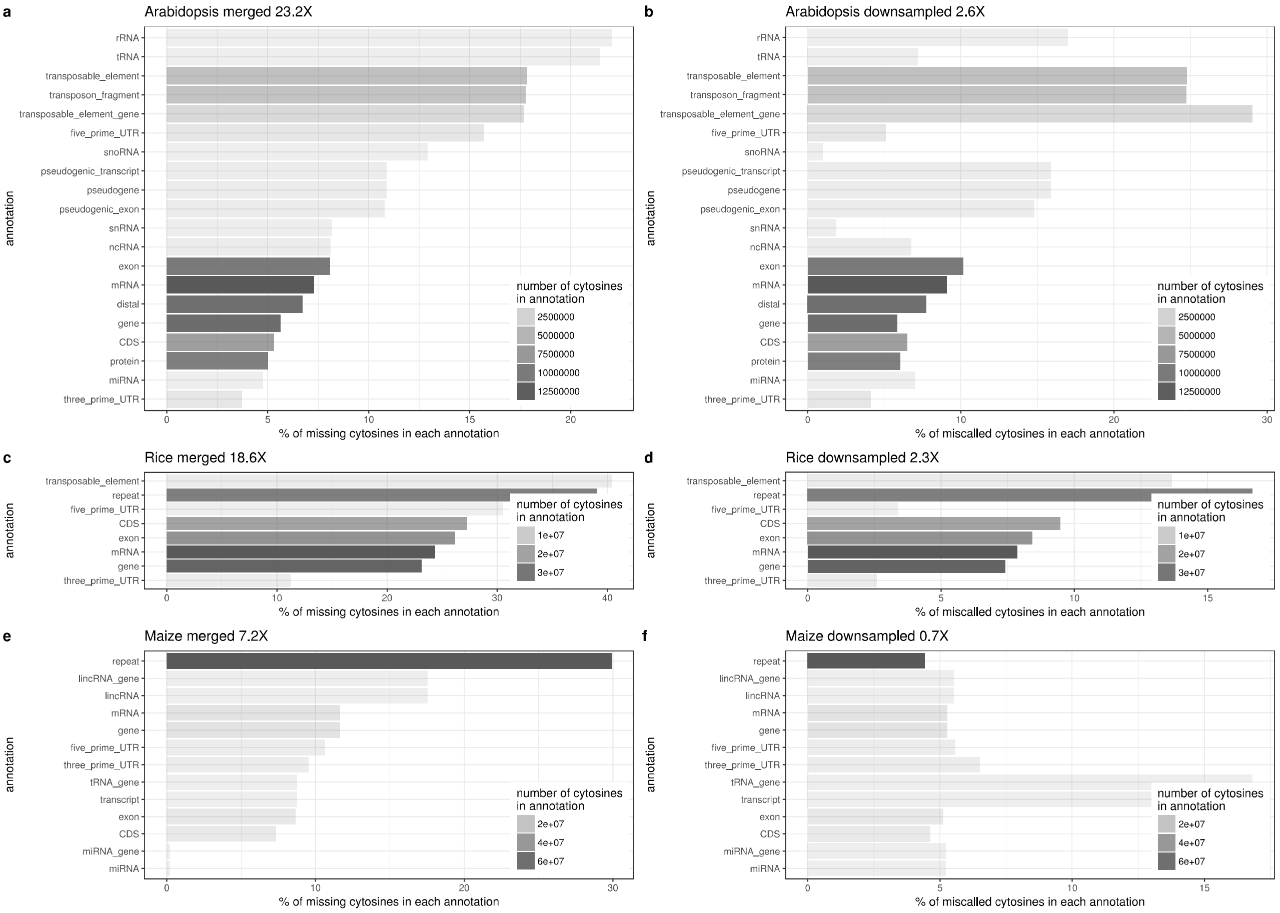

Figure SI-4: Missing and miscalled cytosines per annotation. Left panels show the percentage of missing cytosines per annotation category, right panels show miscalled cytosines in a downsampled dataset. The transparency indicates the number of cytosines in the respective annotation. In all three species, transposable elements and repeats have a higher fraction of missing cytosines compared with genes.

Supplementary Figure 5

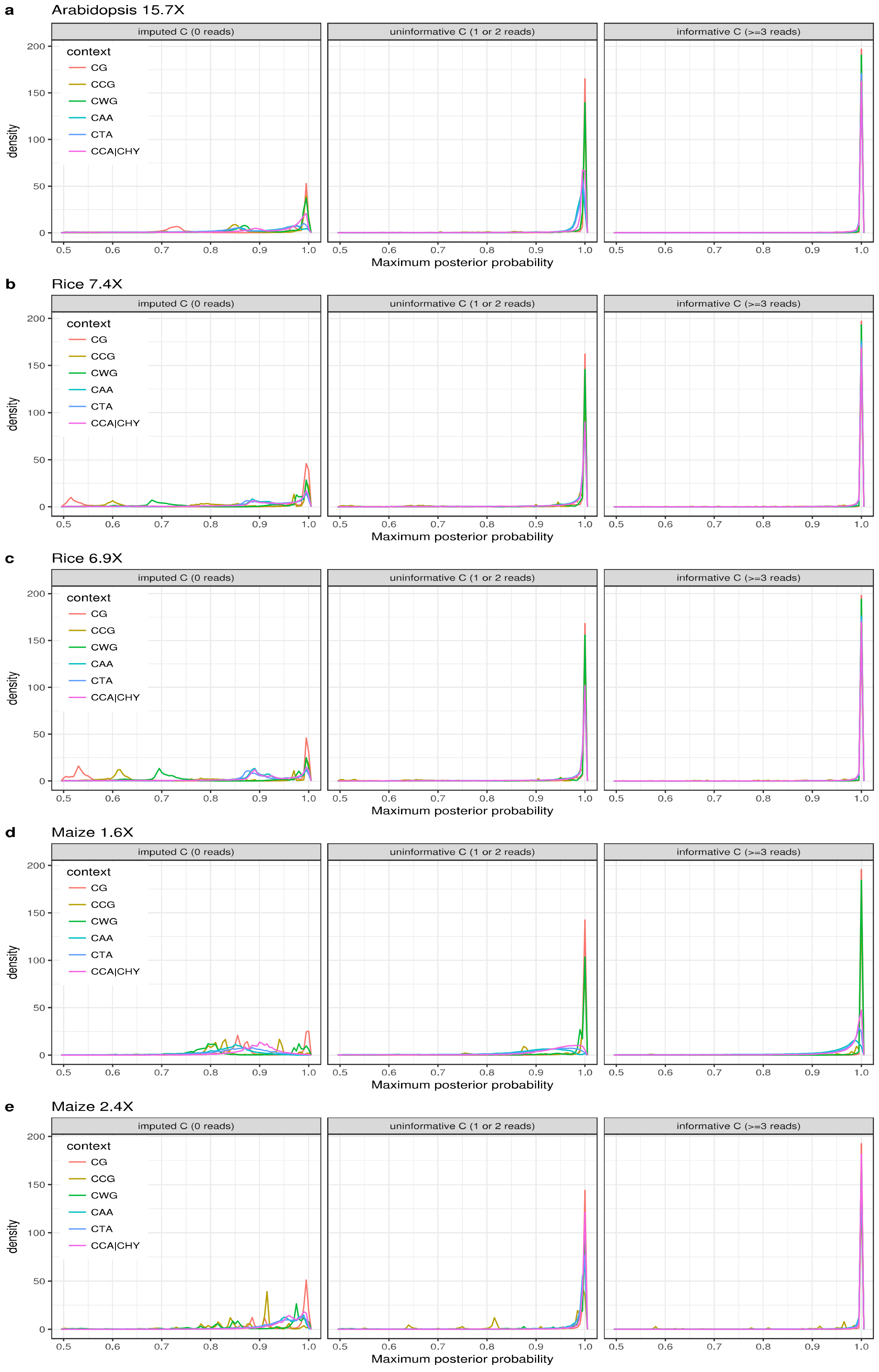

Figure SI-5: Maximum posterior distributions for replicates for imputed cytosines (coverage = 0), uninformative cytosines (coverage = 1 or 2) and informative cytosines (coverage >= 3). The maximum posterior probability, i.e. the confidence in the methylation status calls, is generally lower for sites with less coverage.

Supplementary Figure 6

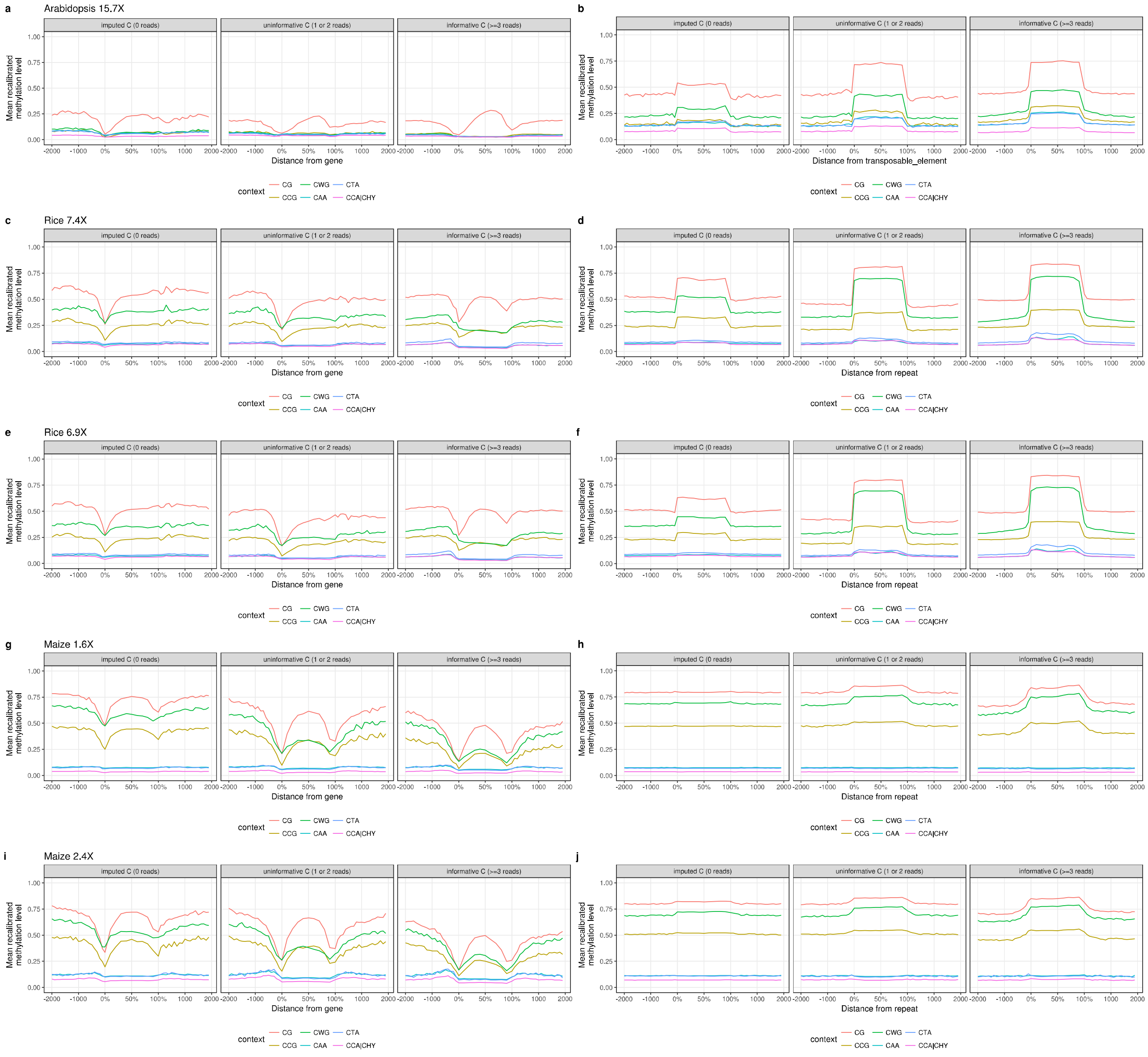

Figure SI-6: Enrichment profiles for replicates for genes (left panels) and transposable elements or repeats (right panels). Sub-panels show the enrichment profiles for imputed (coverage = 0), uninformative (coverage = 1 or 2) and informative cytosines (coverage >= 3).

Supplementary Figure 7

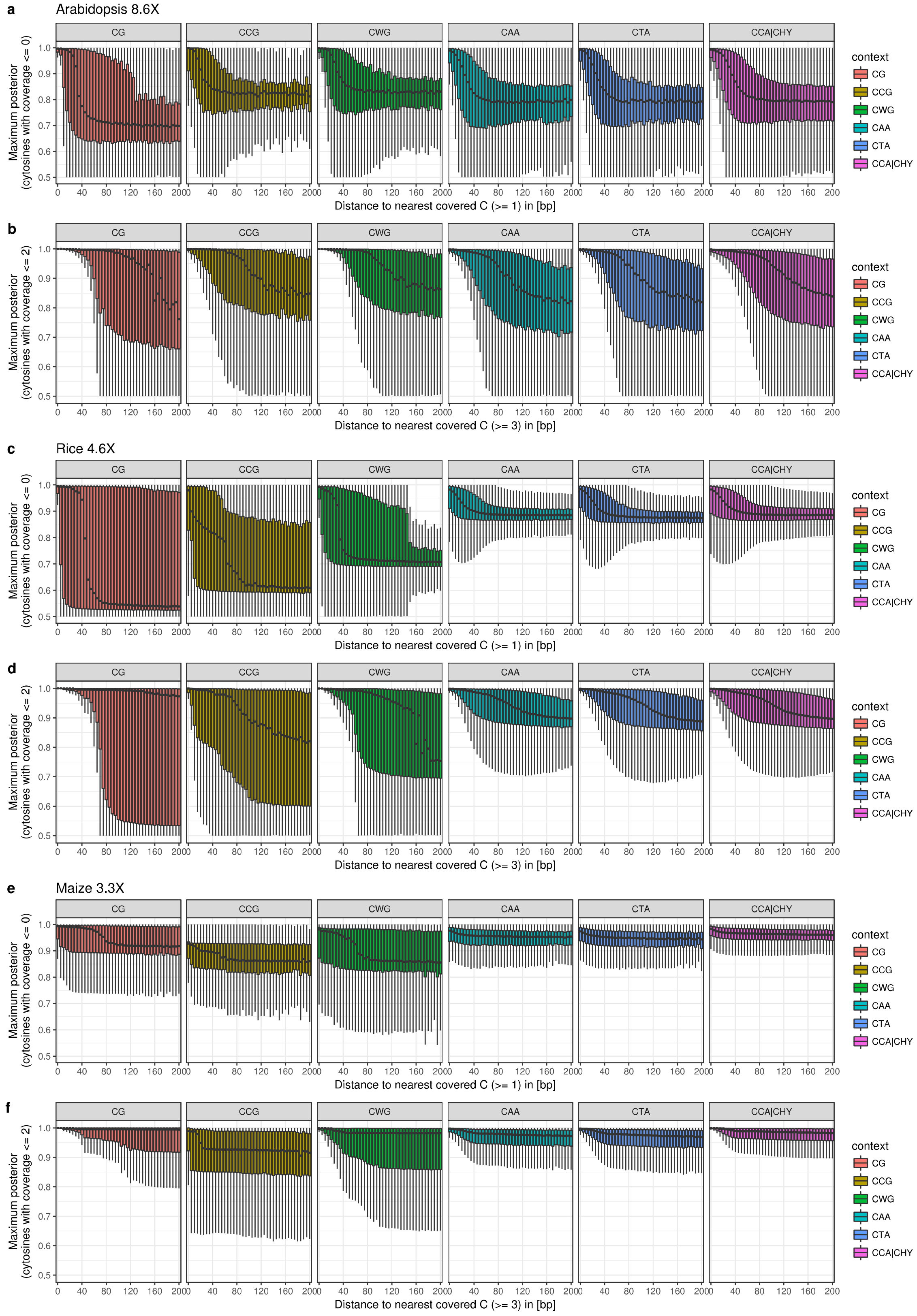

Figure SI-7: The maximum posterior probability (y-axis) is plotted against the distance to the nearest covered cytosine (x-axis). We observe that the maximum posterior probability, i.e. the confidence in the methylation status calls, decays to background levels if the nearest covered cytosine is more than 40-80bp away.

Supplementary Figure 8

a Arabidopsis merged 23.2X

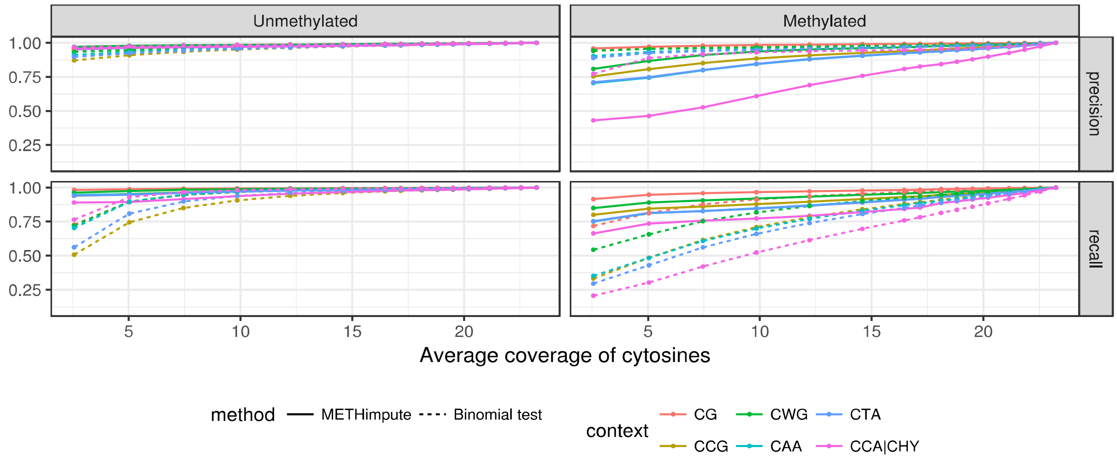

b Rice merged 18.6X

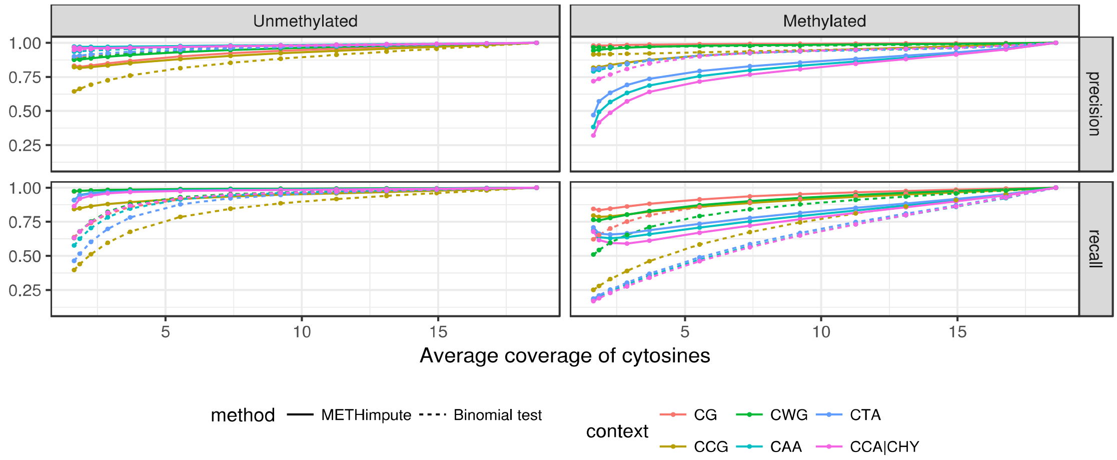

c Maize merged 7.2X

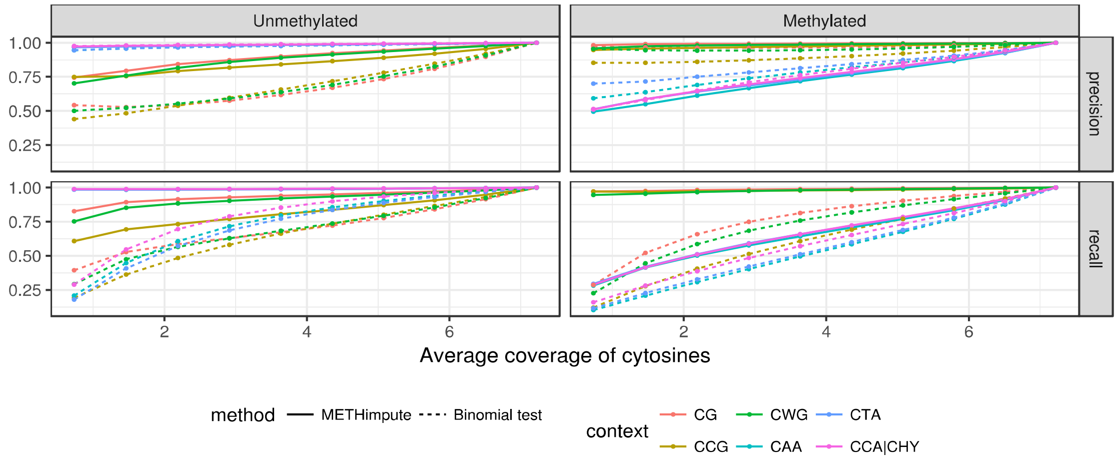

Figure SI-8: Saturation analysis. Precision and recall for the methylation status calls for METHimpute and the binomial test, compared to the full sample, respectively. **(a)** Arabidopsis, **(b)** Rice, **(c)** Maize.

Supplementary Figure 9

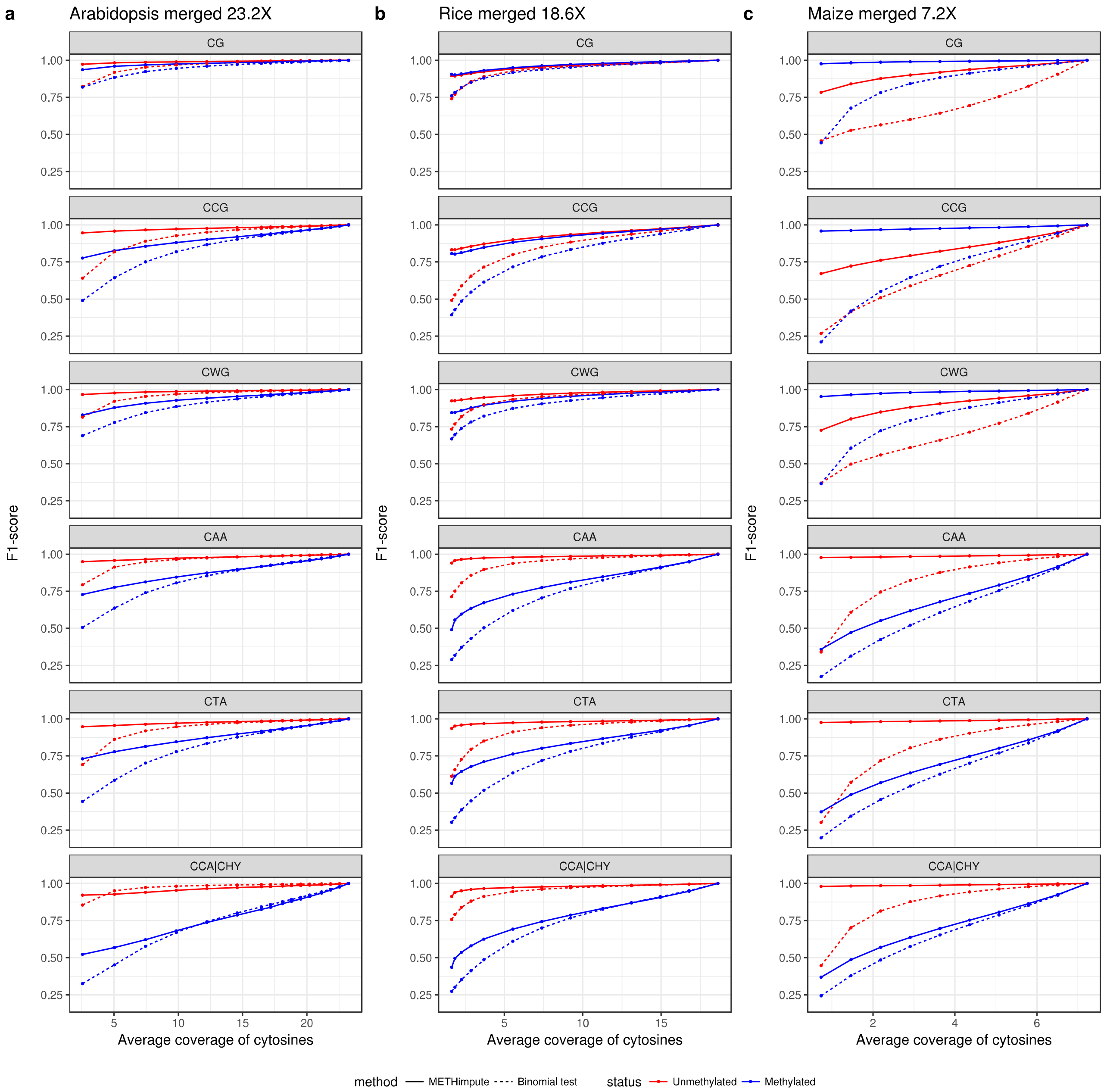

Figure SI-9: Saturation analysis. F1-score for the methylation status calls for METHimpute and the binomial test, compared to the full sample, respectively. F1-score is shown for **(a)** Arabidopsis, **(b)** Rice and **(c)** Maize. Subpanels show the different contexts.

Supplementary Figure 10

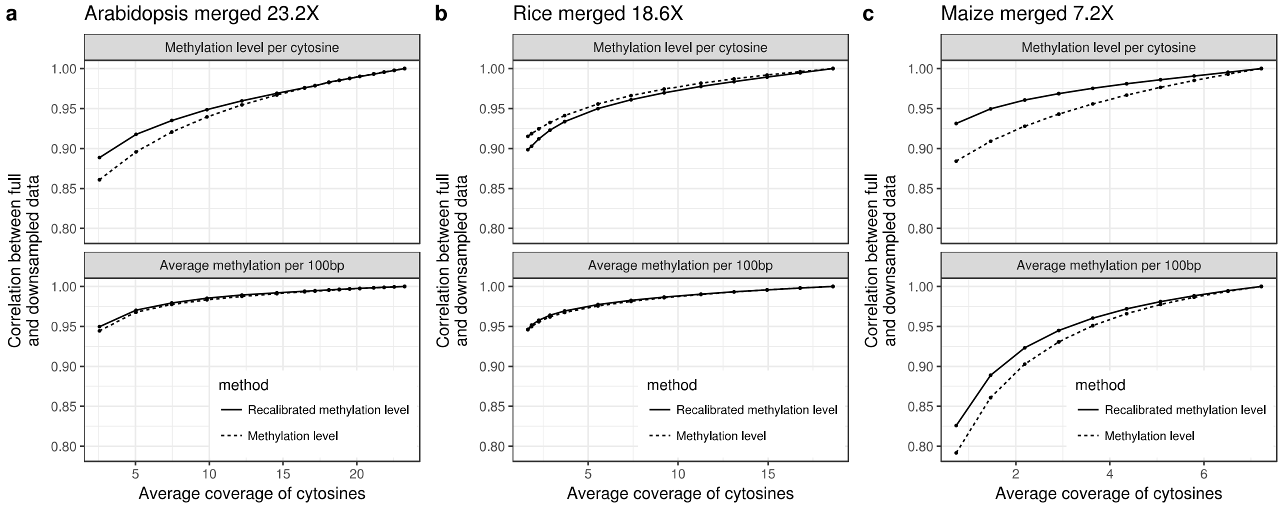

Figure SI-10: Saturation analysis. Correlation between the full and downsampled datasets for original methylation levels and METHimpute recalibrated methylation levels. The correlation is shown for **(a)** Arabidopsis, **(b)** Rice and **(c)** Maize. Top-panels show correlations for individual cytosines, bottom-panels show the correlation for levels averaged (weighted by coverage) over 100bp windows.

Supplementary Figure 11

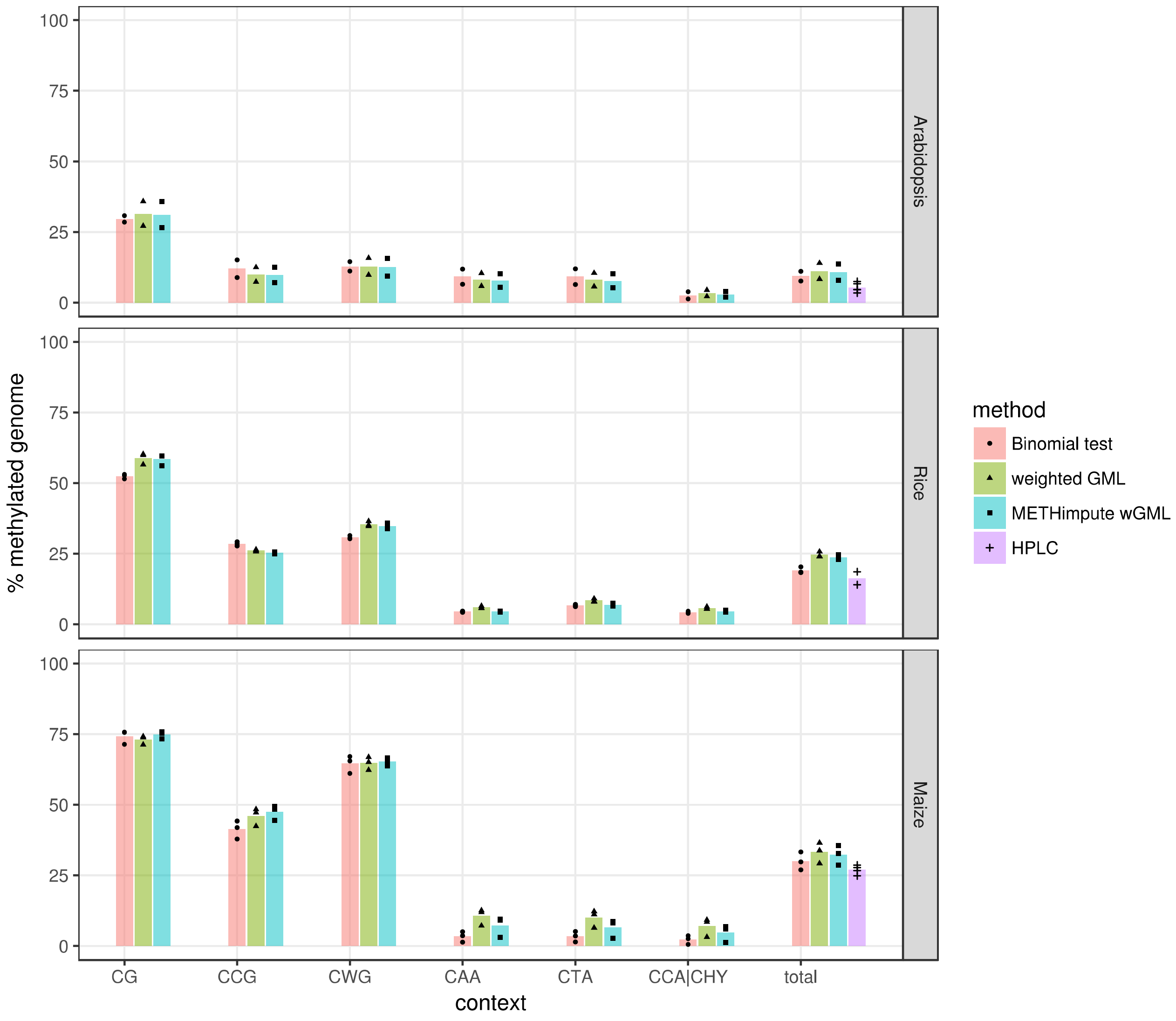

Figure SI-11: Comparison of GMLs. Genome-wide methylation levels (GMLs) calculated by different methods in Arabidopsis, rice and maize. The bar chart indicates the mean methylation level among replicates, dots indicate the methylation level for individual replicates. Differences in GML between HPLC and the other methods are not significant (t-test, $p > 0.05$).

Supplementary Figure 12

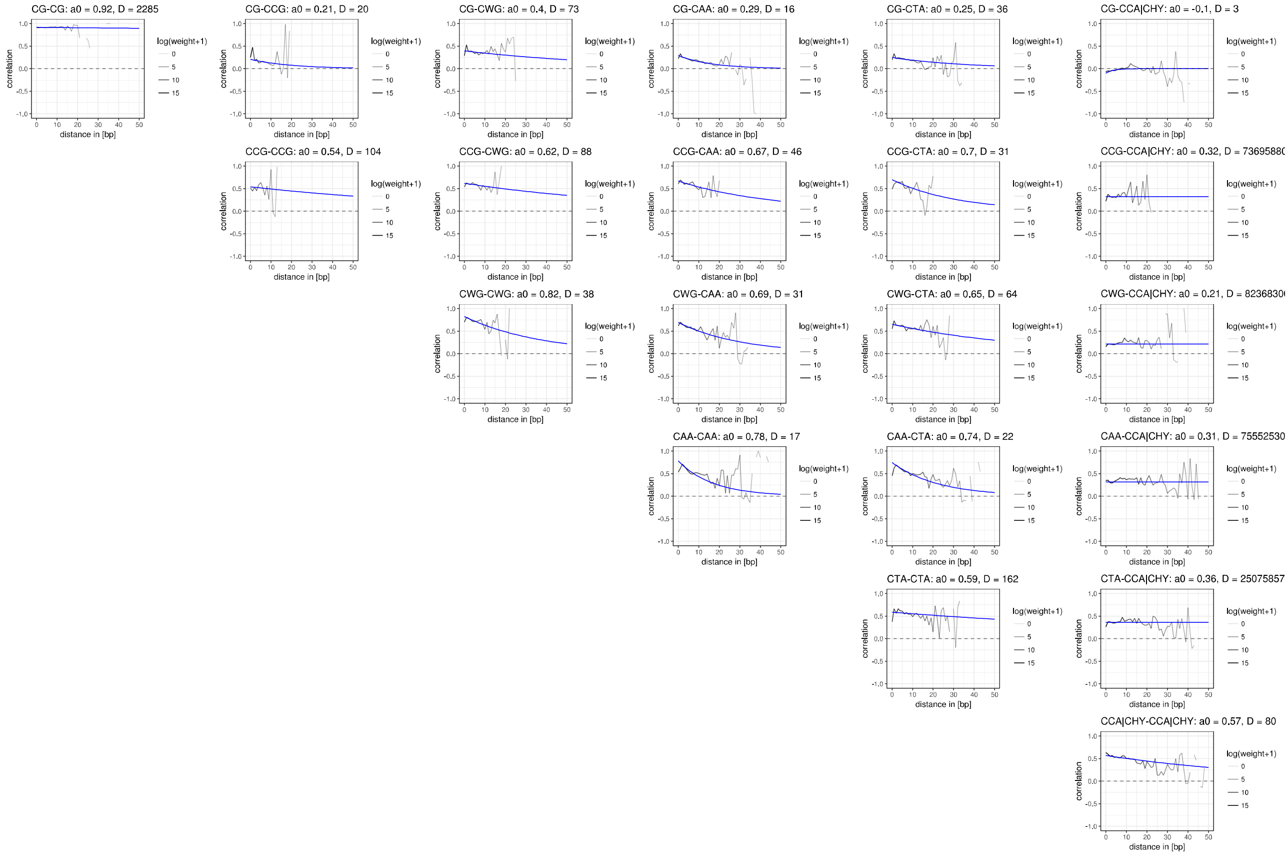

Figure SI-12: Distance correlation. Correlation between the methylation levels of neighboring cytosines, split by context combinations. The distance is defined as the number of base-pairs in between the two neighboring cytosines (without any other cytosines in between). The blue curve is a weighted exponential fit with formula y = a0 * exp(-x/D). The figure shows correlations from sample “Arabidopsis 8.6X”.

References

[1] T. Kawakatsu, S. C. Huang, F. Jupe, E. Sasaki, R. J. Schmitz, M. A. Urich, R. Castanon, J. R. Nery, C. Barragan, Y. He, H. Chen, M. Dubin, C. R. Lee, C. Wang, F. Bemm, C. Becker, R. O'Neil, R. C. O'Malley, D. X. Quarless, The 1001 Genomes Consortium, D. Weigel, M. Nordborg, and J. R. Ecker, "Epigenomic Diversity in a Global Collection of Arabidopsis thaliana Accessions," *Cell*, vol. 166, no. 2, pp. 492–506, 2016.
